# Transcriptome Analysis of the White Body of the Squid *Euprymna tasmanica* with Emphasis on Immune and Hematopoietic Gene Discovery

**DOI:** 10.1371/journal.pone.0119949

**Published:** 2015-03-16

**Authors:** Karla A. Salazar, Nina R. Joffe, Nathalie Dinguirard, Peter Houde, Maria G. Castillo

**Affiliations:** Department of Biology, New Mexico State University, Las Cruces, New Mexico, United States of America; Uppsala University, SWEDEN

## Abstract

In the mutualistic relationship between the squid *Euprymna tasmanica* and the bioluminescent bacterium *Vibrio fischeri*, several host factors, including immune-related proteins, are known to interact and respond specifically and exclusively to the presence of the symbiont. In squid and octopus, the white body is considered to be an immune organ mainly due to the fact that blood cells, or hemocytes, are known to be present in high numbers and in different developmental stages. Hence, the white body has been described as the site of hematopoiesis in cephalopods. However, to our knowledge, there are no studies showing any molecular evidence of such functions. In this study, we performed a transcriptomic analysis of white body tissue of the Southern dumpling squid, *E*. *tasmanica*. Our primary goal was to gain insights into the functions of this tissue and to test for the presence of gene transcripts associated with hematopoietic and immune processes. Several hematopoiesis genes including CPSF1, GATA 2, TFIID, and FGFR2 were found to be expressed in the white body. In addition, transcripts associated with immune-related signal transduction pathways, such as the toll-like receptor/NF-κβ, and MAPK pathways were also found, as well as other immune genes previously identified in *E*. *tasmanica’s* sister species, *E*. *scolopes*. This study is the first to analyze an immune organ within cephalopods, and to provide gene expression data supporting the white body as a hematopoietic tissue.

## Background

It is widely known that all, if not most, organisms establish mutualistic relationships with bacteria. These associations benefit hosts in a variety of ways, which include but are not limited to: metabolic function, disease prevention, antimicrobial peptide production, and nitrogen fixation [1–4]. The association between squid of the genus *Euprymna* and the Gram negative bacterium *Vibrio fischeri* is one of the most studied animal- mutualistic bacterial models. In this host-microbe relationship, the bacteria *V*. *fischeri* colonize the light organ (LO) of the squid, a specialized tissue designed to house the symbiotic bacteria [5]. The squid horizontally acquire these bioluminescent bacteria from the surrounding marine environment [6] which provide these molluscs with downward emitted light to mimic moonlight, and avoid predator detection during their nocturnal activities [5, 7]. In exchange, the symbiont bacteria live in a safe and nutrient-rich environment that favors proliferation.

Much interest exists in understanding the molecular mechanisms that allow the members of mutualistic relationships, such as that of the squid-vibrio, to recognize each other and establish long-term associations. Several studies have suggested that hemocytes, the squid’s phagocyte-like cells, play an important role in the recognition and establishment of the squid-vibrio symbiosis [8, 9]. This has been strongly suggested as hemocytes express a suite of immune genes associated with microbe recognition that are modulated in the presence of *V*. *fischeri* [10, 11]. Previous studies have also shown that squid hemocytes respond to the initial exposure to *V*. *fischeri* by migrating to the juvenile light organ [12, 13], suggesting a chemotactic response to microbial products or components. Additionally, exposure of juvenile squid to *V*. *fischeri* results in differential gene expression of the proteasome-C8 subunit in hemocytes when compared to control animals [13]. This change in C8 gene expression is associated with the characteristic regression of the epithelial surface of the LO, suggesting hemocytes directly respond to the presence of the symbiont and facilitate light organ morphogenesis.

In other cephalopods such as the cuttlefish, *Sepia officinalis*, and the octopus, *Octopus vulgaris*, hemocytes have been described to originate and develop in the white body [14, 15], a soft, multilobed tissue surrounding the optic bundle that connects each of the eyes to the optic lobes. The site of hemocyte production has not been reported in any *Euprymna* species, yet the white body (WB) is thought to serve a similar function as has been proposed in other related cephalopods. Acquisition of molecular data to corroborate the white body as the site of hematopoiesis is an important step in understanding hemocytes, their immune function, and their potential role in the interactions between the host squid and its symbiotic bacteria.

The present study aimed to investigate the function of the white body in *Euprymna tasmanica* by means of transcriptome analysis. WB transcripts were analyzed to assess the gene expression profile of this tissue in adult *E*. *tasmanica* squid. This is the first molecular study to analyze an immune organ in cephalopods and to provide an insight to the biological functions of the mysterious white body.

## Materials and Methods

### Ethics Statement

These studies were conducted with prior authorization from the Institutional Biosafety Committee (IBC) and Institutional Animal Care and Use Committee (IACUC) from New Mexico State University. *Euprymna tasmanica* is not considered an endangered or protected species, thus, no special permits were used during sample collections. Based on protocols previously used with *Euprymna scolopes*, experimental animals were euthanized in artificial seawater (ASW) containing 2% ethanol before dissection, [16–18].

### Animal sampling and antibiotic treatment

Adult *Euprymna tasmanica* squid were collected from Botany Bay, Sydney, Australia and shipped to New Mexico State University. Animals were maintained in 12h/12h light-dark cycle at 18°C in circulating ASW, with a salinity concentration of 34 parts per thousand, following previous published guidelines for *E*. *tasmanica* maintenance [19–22]. Additionally, animals were fed daily with 2–3 live common shore shrimp. Animals used in these experiments were allowed to acclimate for at least four weeks to laboratory conditions. A total of eight adult male squid were used in this study and kept in individual tanks during treatment. Half of the animals were treated with antibiotics (chloramphenicol and gentamycin at a final concentration of 20 μg/mL each) in five-gallon tanks to remove *V*. *fischeri* bacteria from the LO, following a previously published protocol [9]. These animals constituted the “cured” treatment group. To test for the complete absence of bacteria in the LO, the central core (specific site where bacteria reside within the LO) was dissected from each antibiotic-treated animal and homogenized in filter-sterilized seawater. Homogenates were spread on seawater tryptone agar (SWT) plates incubated at 28°C, and later monitored at 24h and 48h. In addition, ASW from the tanks containing cured-animals was filtered using a 0.45 μm sterile filter (Millipore). These filters were then placed in plates containing SWT agar, incubated at 28°C, and subsequently checked at intervals of 24h and 48h for the presence of bacterial colonies. No bacterial colonies were seen in either of the plate treatments. Symbiotic samples were obtained from untreated animals, and their tissues were dissected and stored in RNA later (Life Technologies) and kept at -20°C until needed. At the time of dissection, all animals appeared normal and no other parasites could be detected visually. Despite the fact that animals were inspected for the presence of pathogens and observable signs of infection, the occurrence of visually undetectable parasites or other type of infection was not assessed in the present analysis and the results described in this study are considered to be the major consequence of the experimental treatments. All experimental procedures in this study were performed in duplicates.

### RNA extraction

Adult animals were euthanized in 2% ethanol in ASW, followed by dissection of the white body tissue. White body (WB) tissues were homogenized on ice using a pre-cooled glass homogenizer, and total RNA from cured and symbiotic animals was extracted using TRIzol reagent (Life Technologies). WB-RNA extraction was performed in duplicate using two animals per extraction. Total RNA extracted was diluted with 10 μL of nuclease-free water to quantify and assess quality using a Nanodrop 1000 spectrophotometer (Thermo Scientific). mRNA was purified using the OligoTex directed mRNA mini kit (Qiagen) and following the manufacturer’s general guidelines.

### Fragmentation of mRNA, cDNA library preparations, emulsion PCR, and sequencing

mRNA from cured and symbiotic samples were fragmented and processed following the manufacturer’s protocol (Roche). AMPure beads (Agilent) were used to purify the newly synthesized cDNAs, followed by cDNA-ends repair and RL adaptor ligation (Rapid Library Preparation kit, Roche). Afterwards, residual small cDNA fragments were removed using AMPure beads (Agilent). Both cured and symbiotic white body libraries were quantified against the RL standard (RL cDNA synthesis kit, Roche) using a TBS 380 Fluorometer (Turner Biosystems). Quality and size of the cDNA libraries were verified using High Sensitivity DNA chips on an Agilent 2100 BioAnalyzer. Sequencing was completed using a Roche 454 GS FLX+ sequencer (Genomics Lab, NMSU), and each sample was run on two separate quarters of a plate. Sequences can be accessed on the NCBI SRA (accession number SRP049997).

### Assembly and mapping

Following sequencing, nucleotide sequences were trimmed and assembled using gsAssembler Newbler version 2.6. Only high quality (HQ) reads were trimmed and used in subsequent assembly, mapping, and analysis steps.

### Blast2go and DEseq

All assembled contigs were submitted to Blast2GO (http://www.blast2go.com/b2ghome) to annotate and to assign a gene ontology (GO) classification. Annotation of the assembled contigs was performed using BLASTx algorithm of NCBI non-redundant protein database and InterProScan with an E value of 1.0E-3. Potential differential expression between treatments was estimated using DESeq following vignette’s recommendation for no-biological replicate settings.

### Sequence alignments

Selected transcripts were translated to amino acid sequences and aligned with homolog protein sequences using the software ClustalX 2.1 (http://www.clustal.org/).

## Results and Discussion

### Assembly, annotation, and gene ontology

A summary of the assembly statistics obtained from this *E*. *tasmanica* white body transcriptome is presented in Table 1. A total of 1,053,614 HQ reads were generated, with 495,480 and 558,134 reads for symbiotic and cured samples, respectively. Median read-length was 472 bases for symbiotic and 416 bases for cured samples. HQ reads were trimmed and assembled into 13,446 contigs, from which 2,740 had non-redundant BLASTx hits. Length of the assembled contigs ranged from 102 bp to 7,528 bp, with an average of 672 bp (S1A Fig.). The fact that only 20.37% of the transcripts found annotated BLAST hits suggests that there may be a substantial number of novel genes in *E*. *tasmanica* that remain to be characterized, and emphasizes the need to study cephalopods in more detail to obtain a better understanding of their gene composition and physiology.

**Table 1:** Summary of the white body transcriptome assembly.

Number of sequenced bases	425,644,829
Number of reads	1,053,614
Average read length (bp)	444
Contigs assembled	13,446
Contigs with blast hits	2,740

Blast2GO analysis using the BLASTx algorithm revealed that the transcriptome sequences had between 55% and 65% homology to their corresponding blast hits, with an average of 62.08% homology (S1B Fig.) and an average E value of 1.58E-4 (S1C Fig.). Most blast hits were of the hydrozoan *Hydra magnipapillata*, the silkworm *Bombix mori*, and the Hawaiian bobtail squid *Euprymna scolopes* (S1D Fig.), reflecting the invertebrate phylogeny of squid.

**Fig 1:**
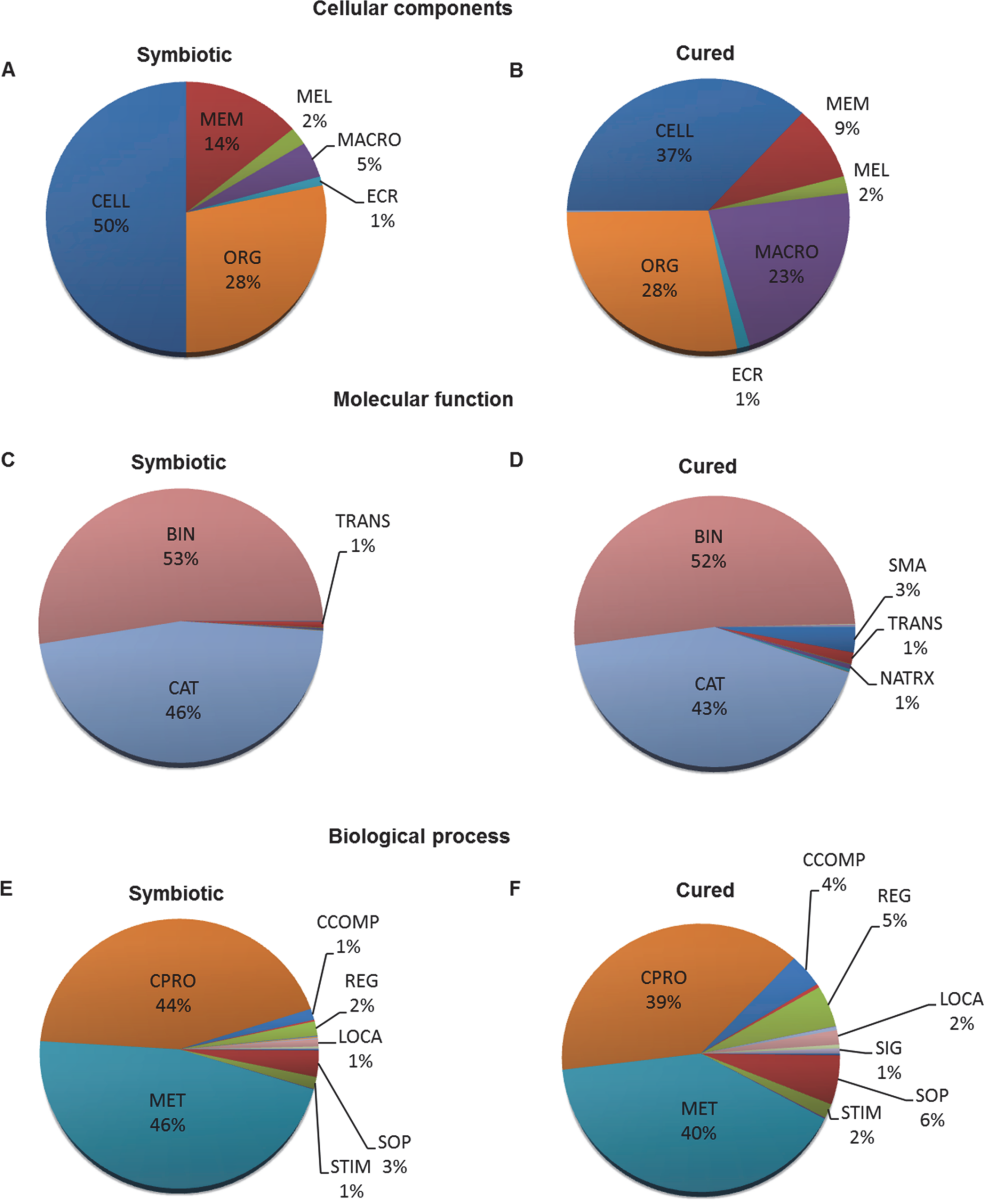
White body transcriptome gene ontology classification. Both symbiotic and antibiotic-treated (cured) treatments showed high percentage of sequences involved principally in enzymatic activity and binding as well as metabolism and general cellular process. (A-B) ***Cell components of symbiotic and cured animals*.** Abbreviations for processes are CELL: cell; MEM: Membrane; MEL: Membrane enclosed lumen; MACRO: Macromolecular complex; ECR: Extracellular region; ORG: Organelle. Transcripts involved in synapse totaled less than 1% and were not included on either chart. (C-D) ***Molecular function of symbiotic and cured animals*.** Abbreviations for processes are SMA: Structural Molecule Activity; TRANS: Transporter Activity; NATRX: Nucleic acid binding transcription activity; CAT: Catalytic activity; BIN: Binding. Transcripts involved in enzyme regulator activity, enzyme carrier activity, molecular transducer activity, translation regulator activity, receptor activity, and protein binding transcription factor activity totaled less than 1% for both treatments and were not included on either chart. Transcripts involved in SMA and NATRX totaled less than 1% for symbiotic organisms and were not included on the chart. (E-F) ***Biological process of symbiotic and cured animals*.** Abbreviations for processes are SOP: Single organism process; STIM: Response to stimulus; MET: Metabolic process; CPRO: Cellular process; CCOMP: Cellular component organization (biogenesis); REG: Biological regulation; LOCA: Localization; SIG: Signaling. Transcripts involved in reproduction, growth, locomotion, development, multi-organism processes, multicellular organismal processes, immune system processes, and biological adhesion totaled to less than 1% in both treatments and were not included in either chart. In symbiotic treatments, transcripts involved in signaling totaled less than 1% and were not included in the chart.

Gene ontology (GO) annotation using the Blast2GO algorithm resulted in 2,229 contigs with a GO functional assignment. The length of the sequences having a GO term ranged between 102 and 6,340 bp (S1E Fig.), and most of these annotations had four or more GO levels assigned to them based on their GO categorization: biological processes, molecular functions, and cell components (S1F Fig.). Gene ontology analysis revealed that most of the sequences could be categorized in binding and enzymatic activity, as well as cellular processes and metabolism (Fig. 1). Furthermore, the number of contigs involved in cellular (44% in symbiotic and 39% in cured) and metabolic processes (46% in symbiotic and 40% in cured) were higher in the symbiotic treatment compared to the cured treatment. Similar patterns were observed in other studies involving beneficial symbioses, such as the symbioses between the tomato *Solanum lycopersicum* and the fungus mycorrhiza *Funneliformis mossae* [23] and between the anemone *Anthopleura elegantissima* with the dinoflagellate *Symbiodinium* [24]. In these studies, it was also found that the presence of the symbionts induced changes in the metabolism of their hosts with tendencies that reflected higher metabolism in colonized compared to non-symbiotic hosts. Preliminary analysis of our data also suggests that the squid physiological demands are different in symbiotic animals, with a higher number of transcripts involved in cellular and metabolic processes in order to maintain and/or regulate the symbionts. Similar results were also observed in colonized *E*. *scolopes*, in which *V*. *fischeri* was found to up-regulate the host metabolism to possibly compensate for a higher-nutrient demand and ensure symbiont maintenance in the LO [25]. Supporting this hypothesis, other studies demonstrated that chitotriosidase, a host enzyme involved in chitin catabolism, was required for successful colonization and maintenance of the symbiont in the LO [25, 26] and was up-regulated in symbiotic animals compared to aposymbiotic squid [27] In contrast, in the present study, the percentage of sequences involved in cellular biogenesis (1.3% in symbiotic and 4% in cured) and biological regulation (1.7% for symbiotic and 5% for cured) were more prevalent in the cured treatment, suggesting an increased gene expression in the absence of bacterial symbionts. Additionally, the percentage of transcripts involved in immune response (5% in cured and 2% in symbiotic) and response to stimulus (39% in cured and 22% in symbiotic) were higher in cured animals than in symbiotic animals (Fig. 1), suggesting a down-regulation of immune genes due to symbiont-mediated responses. It is possible that this down-regulation is necessary for a successful colonization; however, the low replicate number used in this analysis prevents us from formulating any definite conclusions.

### Genes unique to the white body

A total of 71 unique assembled contigs were identified to be expressed solely in the white body when compared to previous LO and hemocyte reports from *E*. *scolopes* [10, 18, 28], with the longest of these being a contig of 919 nucleotides. Blast2GO analysis revealed that none of these contigs presented homology to any other registered sequences in GenBank. Therefore, these sequences were individually analyzed using WU-BLAST algorithm from the European Molecular Biology Laboratory (http://www.ebi.ac.uk/Tools/sss/wublast/nucleotide.html). Nevertheless, no similarities were found after this analysis, suggesting that these transcripts represent novel genes that remain to be characterized. Further analyses need to be done to obtain their full-length coding sequences to characterize them.

### White body involvement in hematopoiesis

One of the goals of this study was to assess the presence of hematopoietic genes in the WB tissue and to confirm previous studies that associate the WB of cephalopods with hematopoietic processes [14, 15]. Hematopoiesis is defined as the biological process of blood-cell formation and differentiation and has been well studied in vertebrates. In these animals, the process starts with self-renewal of multipotent stem cells in the bone marrow that can later differentiate into two diverse progenitor cell lines [29, 30]. Blood-cell differentiation occurs in response to a series of signals initiated by chemical messengers such as cytokines and growth factors, which upon binding to their specific receptors, trigger one of several possible pathways of signal transduction which are known to promote cell division.

Gene ontology analysis of the *E*. *tasmanica* white body transcriptome revealed the presence of eight contigs with homology to genes known to be involved in hematopoiesis in other organisms, including transcripts for the subunit of the cleavage and polyadenylation specificity factor complex (CPSF1), the transcription factor GATA 2, induced myeloid leukemia cell differentiation protein (MCL-1), the ribosomal protein s7, the transcription initiation factor TFIID subunit 3 (TAF3), and the fibroblast growth factor receptor 2 (FGFR2) (Table 2). CPFS1 is implicated in pre-mRNA maturation in zebrafish, and a mutant form was shown to affect maintenance of hematopoietic stem cells in fish embryos [31]. GATA 2, another transcript identified in WB, is an important transcription factor involved in multipotent hematopoietic progenitor proliferation in humans and mice [32–35]. Although the presence of a GATA-3 transcription factor was previously reported in another mollusc, the Pacific oyster *Crassostrea gigas* [36, 37], this is the first time a GATA transcription factor is identified in a cephalopod. The relatedness between these two species was reflected by the results of the alignment between the predicted protein sequences from *C*. *gigas* GATA-3 and *E*. *tasmanica* GATA-2, revealing 47% similarity at the amino acid level. Another transcription factor identified in the WB of *E*. *tasmanica* was TAF3, a key component for early hematopoiesis development in zebrafish embryos and mouse embryotic cells [38, 39]. Equally important in blood cell formation and differentiation are anti-apoptotic genes, such as the proto-oncogene MCL-1, which was also found to be expressed in the white body. In mice, MCL-1 is necessary to prevent apoptosis in early stage hematopoietic progenitors, thus allowing for subsequent differentiation [40]. The presence of a MCL-1 transcript in *E*. *tasmanica* WB suggests that MCL-1 may be involved in the survival of hematopoietic cells in the squid.

**Table 2:** Genes expressed in *E*. *tasmanica* white body tissue related to hematopoiesis.

Putative ID	Organism	Putative function	% of similarity at amino acid level	Accession No.	E-Value
Cleavage and polyadenylation specificity factor subunit 1	*Crassostrea gigas*	mRNA polyadenylation	56%	EKC42064	1.31E-15
Induced myeloid leukemia cell differentiation protein MCL-1-like	*Cricetulus griseus*	Apoptosis regulation	28%	EGW12815	9.22E-15
Transcription factor GATA-2	*Chaetopterus sp*.	Macrophage differentiation	53%	ADC35037	6.43E-24
Ribosomal protein s7	*Crassostrea gigas*	Translational initiation	81%	EKC37696	9.63E-103
Transcription initiation factor TFIID subunit 3	*Columba livia*	Transcription regulation	49%	EMC82588	4.63E-17
Fibroblast growth factor receptor 2	*Crassostrea gigas*	FGFR signaling	69%	EKC18748	2.66E-30
CD109 antigen	*Euprymna scolopes*	Endopeptidase inhibitor	92%	AFV94409	0
Ferritin	*Macrobrachium rosenbergii*	Ion iron transport	38%	AFI54986	8.05E-24

Several fibroblast growth factor receptors (FGFR) found in hematopoietic cells, including FGFR2, have been identified in bone marrow macrophages, granulocytes, B cells, T cells and megakaryocytes in the human erythroleukemia cell line (HEL) suggesting an involvement of FGFR2 in hematopoiesis. Within the present study FGFR2 was identified in the WB. The presence of FGFR2, along with CPSF1, MCL-1, and transcription factors as TAF3 and GATA2 in the white body of the squid, and their known association with hematopoietic processes in vertebrates strongly allude that similar mechanisms may be occurring in phylogenetic distant animals such as cephalopods. Furthermore, the existence of hematopoietic transcripts in adult *E*. *tasmanica* suggests that this process of blood cell formation continues to happen during the lifetime of the squid contrary to *Drosophila melanogaster*, in which hematopoiesis happens only during early stages of development [41].

Interestingly, some transcripts classified as immune related genes were also identified as possible molecules involved in hematopoiesis. For example, CD109, a member of the thioester-containing protein superfamily, is known to be involved in immune system responses in human activated T-cells and platelets [42, 43] and is commonly found in hematopoietic stem cells expressing CD34 [44, 45]. CD109 has also been involved in osteoclastogenesis, a bone marrow cellular process in which the multinucleated osteoclasts differentiate from macrophage cells [46]. Although the direct role of CD109 in these processes has not been defined, the presence and up-regulation of CD109 suggests an important role for this molecule in blood cell formation and differentiation.

Additionally, these findings support the previous reports based on morphological observations implicating the WB in hemocyte formation and development in cephalopods [14, 15]. Although a recent study reported that two types of circulating hemocytes were found in the common octopus, *Octopus vulgaris* [47], so far, only one type of hemocyte has been reported in the squid *E*. *scolopes*, the sister species of *E*. *tasmanica*, [9, 13, 17]. However, more studies need to be done in order to understand the morphological and developmental process of hemocyte formation in *E*. *tasmanica* WB.

### Presence of Immune transcripts in white body

The immune system has evolved to develop components and mechanisms that help organisms to differentiate self from foreign antigens. The innate immune response is characterized by an initial interaction of host pattern recognition receptors or molecules (PRRs, PRMs) with specific microbe-associated molecular patterns (MAMPs) [48–50]. These PRRs are either transmembrane receptors or molecules binding to membrane receptors that trigger a series of signal transduction cascades that ultimately lead to specific immune responses such as inflammation, production of cytokines, cell proliferation, and phagocytosis. Each and all of these immune responses serve to neutralize and/or eliminate pathogenic MAMPs or danger signals that first initiated the reaction. Nonetheless, pathogenic antigens are not the only ones to trigger an immune system response. Most, if not all animals, interact and associate with beneficial microorganisms, leading to an immune interaction between host and non-pathogenic organisms [51–56]. What we do not yet understand, is which molecular mechanisms are involved in host-beneficial microbe recognition and how the immune system recognizes these foreign cells as non-pathogenic. Since the squid-vibrio model offers an excellent model for the study of animal host-beneficial microbe interactions, another important goal in this transcriptomic study was to expand our knowledge of immune-related molecules present in the squid by analyzing immune-related transcripts in the WB of *E*. *tasmanica*.

**Fig 2:**
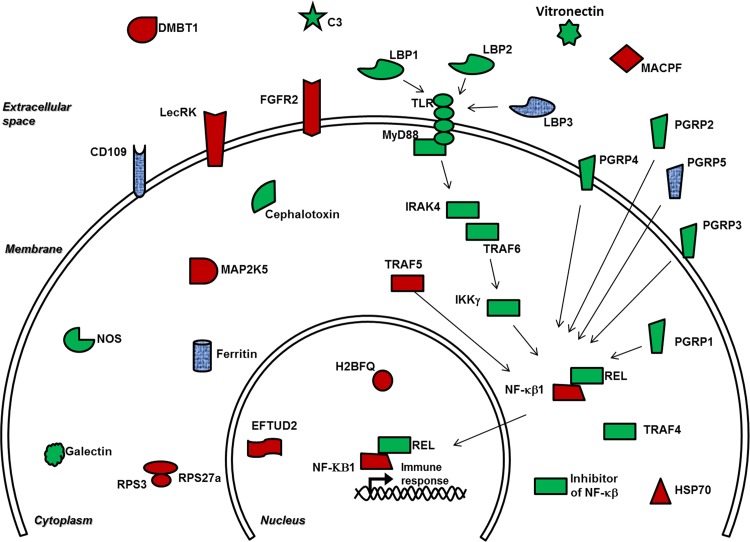
Immune genes expressed in *Euprymna sp*. The shapes in red indicate genes discovered only in *E*. *tasmanica* white body. Genes previously reported in *E*. *scolopes* are in green. Blue shapes are immune genes identified in both species. Abbreviations are as follows: C3: complement component 3; DMBT1: deleted in malignant brain tumor 1; EFTUD2: elongation factor Tu GTP binding domain containing 2; FGFR2: fibroblast growth factor receptor 2; H2BFQ: histone h2b type 2-e-like; HSP70: heat shock protein 70; IKKγ: Iκβ kinase γ; IRAK4: interleukin-1 receptor-associated kinase 4; LecRK: lectin receptor kinase; LBP1: lipopolysaccharide binding protein 1; LBP2: lipopolysaccharide binding protein 2; LBP3: lipopolysaccharide binding protein 3; MAP2K5: dual specificity mitogen-activated protein kinase kinase 5-like; MACPF: mac/perforin domain containing protein; MyD88: myeloid differentiation primary response 88; NF-κβ1: nuclear factor NF-kappa-β p105 subunit; NOS: nitric oxide synthase; PGRP1: peptidoglycan recognition protein 1; PGRP2: peptidoglycan recognition protein 2; PGRP3: peptidoglycan recognition protein 3; PGRP4: peptidoglycan recognition protein 4; PGRP 5: peptidoglycan recognition 5; REL: proto-oncogene c-Rel; RPS3: Ribosomal protein s3; RPS27a: ubiquitin-40s ribosomal protein s27a; TLR: toll-like receptor;TRAF4: tumor necrosis factor—receptor- associated factor 4-like; TRAF5: tumor necrosis factor- receptor- associated factor 5-like; TRAF6: tumor necrosis factor—receptor- associated factor 6-like.

Several immune related transcripts were found to be expressed in *E*. *tasmanica*’s WB. Transcripts involved in the NF-κβ and Toll signaling pathway were identified, including a nuclear factor NF-kappa-β p105 subunit and a lipopolysaccharide binding protein 3. The toll/NF-κβ pathway is activated after MAMPs interact with their specific ligands and trigger an innate immune response by initiating transcription of effector molecules. These findings corroborate previous studies identifying several components of the NF-κβ pathway in *E*. *scolopes* juveniles and adult hemocytes [10, 11, 57]. Among the immune-related molecules previously identified in *E*. *scolopes*, several pattern recognition proteins were found, such as the lipopolysaccharide binding proteins (LPB) 1–3, and the peptidoglycan recognition proteins (PGRP) 1–5 (Fig. 2). Since *E*. *scolopes* and E. *tasmanica* are sister species, their immune-related molecules are likely to be functionally similar and present high sequence homologies. Additional transcripts for several other signaling molecules were identified in the WB, including TNF-receptor-associated factor 5-like (TRAF5), dual specificity mitogen-activated protein kinase kinase 5-like (MAP2K5), and ubiquitin-RPS27a and RPS3 (Table 3).

**Table 3:** Genes expressed in *E*. *tasmanica* white body involved in immune response.

Putative ID	Organism	Putative function	% of similarity at amino acid level	Accession No.	E-Value
Ubiquitin-40s ribosomal protein s27a	*Crassostrea gigas*	NF-κβ & MAPK signaling	86%	AFI80900	1.23E-74
Ribosomal protein s3	*Osmerus mordax*	NF-κβ signaling	56%	EKC30814	3E-144
Elongation factor Tu GTP binding domain containing 2	*Culex quinquefasciatus*	mRNA splicing regulator	77%	XP_001841734	6E-6
Dual specificity mitogen-activated protein kinase kinase 5-like	*Anas platyrhynchos*	MAPK & NF-κβ signaling	57%	EOA93973	4.43E-36
Nuclear factor NF-kappa-β p105 subunit	*Sepia officinalis*	NF-κβ signaling	63%	AEE87261	2E-65
Fibroblast grow factor receptor 2	*Crassostrea gigas*	FGFR signaling	69%	EKC18748	2.66E-30
Histone h2b type 2-e-like	*Mus musculus*	Response to Gram-negative bacterium	86%	NP_835586	1.61E-53
Lectin receptor kinase	*Arabidopsis thaliana*	Plant immune defense	42%	AEE79957	4E-6
Heat shock protein 70	*Azumapecten farreri*	Response to stress	91%	AAO38780	0
MACPF domain containing protein	*Mytilus galloprovincialis*	Cell death	45%	AEK10751	4.0E-41
CD109	*Euprymna scolopes*	Endopeptidase inhibitor	92%	AFV94409	*0*
TNF receptor-associated factor 5-like	*Saccoglossus kowalevskii*	NF-κβ signaling	48%	XP_006824642	3.96E-10
Deleted in malignant brain tumors 1 partial	*Crassostrea gigas*	PRR^1^	54%	EKC27306	2.62E-17
Peptidoglycan recognition protein 5 precursor	*Euprymna scolopes*	Defense response to Gram-positive bacterium	87%	AIR71819	8E-104
Ferritin	*Macrobrachium rosenbergii*	Ion iron transport	38%	AFI54986	8.05E-24
Lipopolysaccharide binding protein 3	*Euprymna scolopes*	Response to Gram-negative bacteria	88%	AEL03862	4E-167

^1^ Pattern recognition receptor

MAP2K5 is an effector molecule involved in the MAPK and NF-κβ intracellular signaling cascade that leads to cell differentiation, proliferation and angiogenesis. This pathway is triggered by mitogens, cytokines, and reactive oxygen species (ROS), which in turn activate MAP2K7 leading to cell differentiation [58].

TRAF5 protein mediates the signal transduction of NF-κβ pathway via TNF receptor activation [59, 60]. This adaptor protein leads to the activation of NF-κβ transcription factor and posterior immune and inflammatory responses [61]. *Listeria monocytogenes* infection in mice leads to TRAF5-dependent T cell activation and proliferation [62], supporting the importance of TRAF5 in the immune system response to bacteria. The presence of TRAF5 was already identified in a transcriptome analysis of *O*. *vulgaris* hemocytes [63], indicating the presence of TRAF5 in both octopus and squid.

The elongation factor Tu GTP binding domain containing 2 (EFTUD2) is a component of the splicesosome complex involved in pre-mRNA processing [64, 65]. Down regulation of EFTUD2 affects *Caenorhabditis elegans* survival after Gram negative bacterial exposure and EFTUD2 overexpression increased the immune response of murine macrophage cells exposed to lipopolysaccharide. In these same cells, it was also shown that EFTUD2 regulates mRNA splicing of the adaptor MyD88, which forms part of the Toll-like signaling pathway [66].

Interestingly, a transcript containing the membrane attack complex/perforin domain (MACPF) was also found expressed in the squid’s WB. The MACPF domain is present in the protein perforin, which is released upon activation by cytotoxic T cells [67]. Additionally, the MACPF domain is commonly found in proteins that form the membrane attack complex (MAC), the complement components C6, C7, C8, and C9. These MAC-containing proteins have been well described in vertebrates, and are responsible for making pores in the cell membrane of microorganisms that ultimately induce lysis. Proteins containing MACPF domains have also been reported in other molluscs such as the Mediterranean mussel *Mytilus galloprovincialis* [68] and the aquatic snail *Pomacea canaliculata* [69]. In the Mediterranean mussel, bacterial challenge produced an increase in the expression of MACPF in gills and hemocytes, indicating a possible immune function for this molecule in mussels [68]. In the case of *E*. *tasmanica*, the identified transcript contains the conserved signature motif present in all previously reported MACPF proteins (S2A Fig.), but further studies need to be performed to verify its possible immune function.

A transcript coding for deleted in malignant brain tumor 1 protein (DMBT1) was also identified in the *E*. *tasmanica* transcriptome. DMBT1 has been reported to act as a possible pattern recognition receptor in the mucosa of mammals by binding to different bacteria [70–72]. Bikker and colleagues demonstrated that the scavenger receptor cysteine rich domain (SRCR) present in DMBT1 is responsible for the reported bacterial binding function [72]. This SRCR domain was identified in *E*. *tasmanica* DMBT1 contig, and contains the VEVLXXXXW motif reportedly involved in bacterial binding [72] with only one amino acid substitution (methionine instead of leucine; S2B Fig.). Additionally, it has been shown that in the presence of LPS, DMBT1 inhibits NF-κβ activation via TLR4, thus preventing an inflammatory response. Furthermore, LPS and TNFα stimuli lead to DMBT1 up-regulation, suggesting there is a possible role of DMBT1 in the prevention of bacterial infection [73]; however, further work needs to be done in *E*. *tasmanica* to understand the potential involvement of DMBT1 in the squid-vibrio symbiosis.

In host-microorganism associations, iron takes an important role. Ferritin and transferrin are two proteins involved in cellular iron homeostasis [74] that were expressed in *E*. *tasmanica’*s WB. Both proteins were also previously identified in *E*. *scolopes* [28] and are involved in the squid-vibrio symbiosis [27, 75]. Kremer and colleagues indicated the importance of ferritin in this symbiosis when they showed that ferritin expression was up-regulated in symbiotic animals when compared to non-symbiotic juveniles. However, in the obligate symbiosis between the bacteria *Wolbachia* and the wasp *Asobara tabida*, ferritin was up-regulated in aposymbiotic wasps in comparison with symbiotic wasps, indicating that *Wolbachia* possibly regulates ferritin expression in the host [76]. Additionally, ferritin has been associated in hematopoiesis. In HL60 promyelocytic leukemia cells, macrophage, and neutrophil differentiation induce ferritin up-regulation [77]. A similar up-regulation was reported in the THP1 monocytic cell line after induction of macrophage differentiation [78].

Peptidoglycan recognition proteins (PGRPs) are pattern recognition receptors that bind to bacterial peptidoglycan (PGN) and in some cases, lead to PGN degradation [79]. Four PGRPs have been previously identified in the LO of *E*. *scolopes*, PGRP 1–4 [57], and are involved in successful LO colonization [80, 81]. Further studies identified a fifth PGRP in *E*. *scolopes* hemocytes [10], which was found to be up-regulated in mRNA of symbiotic animals compared with antibiotic-treated squids, suggesting a role for this protein in the maintenance of the symbiosis. The *E*. *tasmanica* PGRP expressed in WB was identified as a PGRP5 after AA alignment with *E*. *scolopes* PGRPs, showing 87% identity (S2C Fig.), suggesting that these two molecules are orthologs. The identified transcript of Et-PGRP5 was found to code for the complete protein, and a detailed analysis revealed the presence of the characteristic substrate-binding site involved in MAMP recognition [82] and the amidase catalytic site involved in peptidoglycan hydrolysis [79, 83]) (S2 Fig.). The presence of these conserved residues in Et-PGRP5 suggests that PGRP5 could be involved in bacterial PGN recognition and degradation.

**Fig 3:**
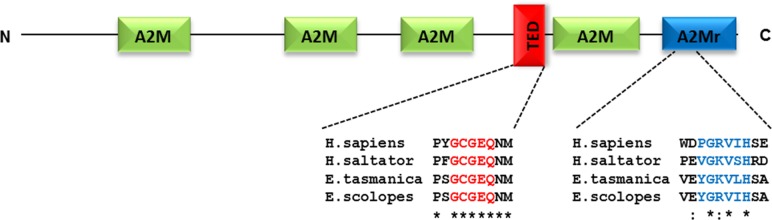
Domain composition of *E*. *tasmanica* CD109. Et-CD109 domain organization recognized by the Conserved Domain Search Service from NCBI. EtCD109 consists of four macroglobulin domains of alpha-2-macroglobulin (A2M) family of proteins which are indicated in green. The thioester domain (TED) found in all members of the TEP superfamily is shown in red. The alpha-2-macroglubulin receptor (A2Mr) motif is marked in blue. Organisms common names and GeneBank accession numbers: *Homo sapiens* (human) CD109 (AAN78483); *Harpegnathos saltator* (Jerdon’s jumping ant) CD109 (EFN86807); *Euprymna scolopes* (bobtail squid) TEP (AFV94409).

The thioester-containing proteins are known to have important functions in the immune system, such as opsonization and clearance of antigens [84, 85]. Some of these TEP components are the well-studied proteins of the complement system (C3, C4, and C5), others serve as protease inhibitors such as the alpha-2-macroglobulin [86–89]. Soluble members of the thioester-containing protein (TEPs) superfamily were previously identified in *E*. *scolopes* squid, including two TEPs, an alpha-2-macroglobulin, and C3, which is the central component of the complement system [10, 28, 90]. In the present transcriptome, a CD109 antigen, an additional member of the TEPs superfamily was identified. CD109 antigen is a membrane bound protein found in activated platelets and T-cells [42, 43], and it was recently recognized as a closely related protein to alpha-2-macroglobulin (A2M) [43]. Et-CD109 contains the conserved thioester signature motif GCGEQ present in all members of TEP superfamily (Fig. 3). In addition to the thioester domain (TED), Et-CD109 contains four alpha-2-macroglobulin (A2M) domains and an alpha-2-macroglobulin receptor (A2Mr) domain. Downstream of the TED, Et-CD109 contains the thioester-reactivity-defining hexapeptide motif PGRVIH. The presence of a histidine residue at the end of the motif suggests this protein favors formation of covalent bonds with hydroxyl groups in target carbohydrate and protein molecules [87, 91, 92]. The presence of this histidine residue is associated more often with CD109- and complement-members of the TEP superfamily and has served to differentiate molecules from A2Ms or insect TEPs (iTEPs). CD109 was originally identified as a cell surface antigen in a myelogenous leukemia cell line (KG1a) [42] and bound to the plasma membrane via a glycosylphophatidylinositol (GPI)-anchor. Analysis using the big-PI predictor site (http://mendel.imp.ac.at/sat/gpi/gpi_server.html) identified a potential GPI-anchor site in Et-CD109 on the serine residue number 1378. Similar results were obtained analyzing human CD109 [43]. An almost identical transcript to *E*. *tasmanica* CD109 (Et-CD109) was previously identified by our group in *E*. *scolopes tissues* (GenBank accession number AFV94409) and was initially annotated as a thioester-containing protein. However, upon detailed analysis, it was re-annotated as a CD109 homologue. Amino acid alignment of Et-CD109 and *E*. *scolopes* CD109 indicated that the sequences are 95% identical but further analysis of this *E*. *tasmanica* contig is needed to confirm the accuracy of the sequence and define its annotation.

Gene ontology analysis allowed us to identify additional immune-related proteins that were not previously described in *Euprymna tasmanica* (Table 3 and Fig. 2). These include nuclear factor NF-κβ p105 subunit (NF-κβ1), lectin receptor kinase (LecRK), fibroblast growth factor receptor 2 (FGFR2), and heat shock protein 70 (HSP70). A comparison of immune-related transcripts in the WB of symbiotic *versus* cured animals showed differences suggesting a minor increase in this type of transcripts in cured hosts (Table 4). Similar results were previously reported for *E*. *scolopes*, showing for example, a down-regulation of *E*. *scolopes* nitric oxide and nitric oxide synthase (NOS) levels (usually produced as defense against pathogens) in the presence of *V*. *fischeri* [93, 94], and the ability of the bioluminescent symbiont to regulate the expression of *E*. *scolopes* immune genes over time and symbiont abundance [95]. Altogether, these findings suggest a possible trend of a lower expression of immune genes in symbiotic animals compared to cured animals; nevertheless further expression analysis is necessary to confirm these findings.

**Table 4:** *E*. *tasmanica* white body transcripts involved in immune response and their expression between treatments.

Putative ID	Symbiotic treatment	Cured treatment
Elongation factor Tu GTP binding domain containing 2	*+*	
Dual specificity mitogen-activated protein kinase kinase 5-like	*+*	
Nuclear factor NF-kappa-β p105 subunit	*+*	*+*
Fibroblast grow factor receptor 2	*+*	
Lectin receptor kinase	*+*	
Heat shock protein 70		+
MACPF domain containing protein		+
CD109		+
TNF receptor-associated factor 5-like		+
Deleted in malignant brain tumors 1 partial		+
Peptidoglycan recognition protein 5 precursor		+
Ferritin		+
Lipopolysaccharide binding protein 3	*+*	

The presence of immune-related genes in *E*. *tasmanica* WB strongly suggests that this tissue can be considered a primary immune organ in the squid and possibly other cephalopods. The presence of immune genes could indicate that white body hemocytes are already expressing some of their characteristic immune-related genes, and that these markers could be used as indicators of their development and/or activation stage. Furthermore, the finding of immune genes normally expressed upon microbial challenge, could represent the presence of immune activated hemocytes transitioning through the WB and potentially be involved in naïve cell training or maturation. A previous study with *E*. *scolopes* hemocytes showed that curing adult animals with antibiotics led to a significant up-regulation of hemocyte binding and phagocytosis of *V*. *fischeri* [9]. This study suggested the presence of some form of hemocyte immune priming mechanism, where naïve hemocytes are “trained” to recognize or tolerate the symbiont. Immune priming has been previously reported in different invertebrate species. For example, in the snail *Biomphalaria glabrata*, secondary exposure to the parasite *Schistosoma mansoni* prevented a secondary infection [96]. Immune priming was also observed in the fruit fly *Drosophila melanogaster* after secondary infection with *Streptococcus pneumonia*, in which phagocytes played an important role [97]. Nevertheless, additional studies in *E*. *tasmanica* are required to understand and compare the characteristics and behavior of WB-hemocytes and hemocytes in circulation or localized in other tissues, as well as the possible influence of symbiotic *V*. *fischeri* on the development and maturation of immune cells.

The finding of constitutively expressed immune transcripts in the WB of the squid *E*. *tasmanica*, an organ that is not in direct contact with any known microorganism is intriguing and raises more questions regarding the development of immune responses in invertebrates. Future studies aimed to understand the life history of hemocytes from their origin to their effector function may help to clarify the role of the WB in *E*. *tasmanica*.

## Conclusions

The symbiotic relationship between *E*. *tasmanica* and *V*. *fischeri* is likely the result of a regulated interchange of molecular messages between the two organisms. This molecular “conversation” potentially helps to modulate physiological processes in the squid host to ensure the success and maintenance of the symbiont colonization. Very little is known about the immune system of *Euprymna* squid, and of cephalopods in general. Nonetheless, previous reports have suggested a possible role of the white body as a hematopoietic and immune organ (for a more detailed study in cephalopods white body morphology see [15]). Thus, the main objective of the present work was to obtain molecular evidence for these reported functions by performing a transcriptome analysis of the white body from the squid *E*. *tasmanica*. We were able to confirm the presence of both hematopoietic- and immune-related transcripts in the squid’s WB. Several immune genes previously reported in the LO and hemocytes of *E*. *scolopes* have been also found expressed in the WB of *E*. *tasmanica*. The high percentage identities found among the immune transcripts of these two species suggest that they have a very similar immune system and it authenticates the use of these two squid species in comparative studies. In addition, we got a first glance of potential systemic effects of the presence of *V*. *fischeri* bacteria in other tissues besides the LO although additional analysis is needed.

One of the initial responses animals show after immune activation is an increase in the production of immune cells [98–100]. The identification of immune transcripts, in addition to the expression of several genes known to be involved in hematopoiesis, support the hypothesis that the white body is the primary site of hemocyte production and development.

The effects that *V*. *fischeri* colonization have on gene expression in the white body are currently being investigated by our group. These studies will provide a better understanding of the systemic effects upon establishment of beneficial symbiosis, and more specifically in the hematopoietic process in cephalopods.

## Supporting Information

S1 Fig Blast2GO distribution of *Euprymna tasmanica* white body transcriptome assembled contigs. The transcriptome dataset was analyzed with Blast2GO software. (A) Length distribution of the assembled contigs. (B) Sequence similarity corresponding to BLAST hits. (C) Species distribution using BLASTx database. (D) Expected value distribution of sequence hits. (E) Number of GO terms assigned by sequences length. (F) Amount of GO levels assigned to annotated contigs based on gene ontology categorization.
(TIF)

S2 Fig Multiple sequence acid alignment of selected domains from immune molecules identified in *E*. *tasmanica*. Newly identified immune transcripts in *E*. *tasmanica* were aligned with their close homologs protein sequences using ClustalX 2.1. A) Alignment of MAC perforin motifs (MACPF) is indicated in red. B) Comparison of DMBT1 protein sequences. VEVLXXXXW motif from the scavenger receptor cysteine rich domain (SRCR) in DMBT1 is marked in blue. C) *E*. *tasmanica* and *E*. *scolopes* peptidoglycan recognition protein 5 (PGRP5) comparison. The amino acids involved in the catalytic amidase site are highlighted in yellow. The arrowheads indicate substrate binding sites. For all alignments, asterisks indicate the conserved amino acids among proteins. GenBank accession numbers used in this figure: MACPF: Mediterranean mussel, *Mytilus galloprovincialis* (AEK10751); Aquatic snail, *Pomacea canaliculata* (P0C8G6); DMBT1: Pacific oyster, *Crassostrea gigas* (EKC27306); Human, *Homo sapiens* (NP_015568); PGRP5: Bobtail squid, *Euprymna scolopes* (AIR71819).
(TIF)
