# Supplementary figures and images for: Transcriptome Analysis of the White Body of the Squid Euprymna tasmanica with Emphasis on Immune and Hematopoietic Gene Discovery
Source: PLoS One. 2015 Mar 16;10(3):e0119949. doi: 10.1371/journal.pone.0119949 (PMC4361686; doi:10.1371/journal.pone.0119949)

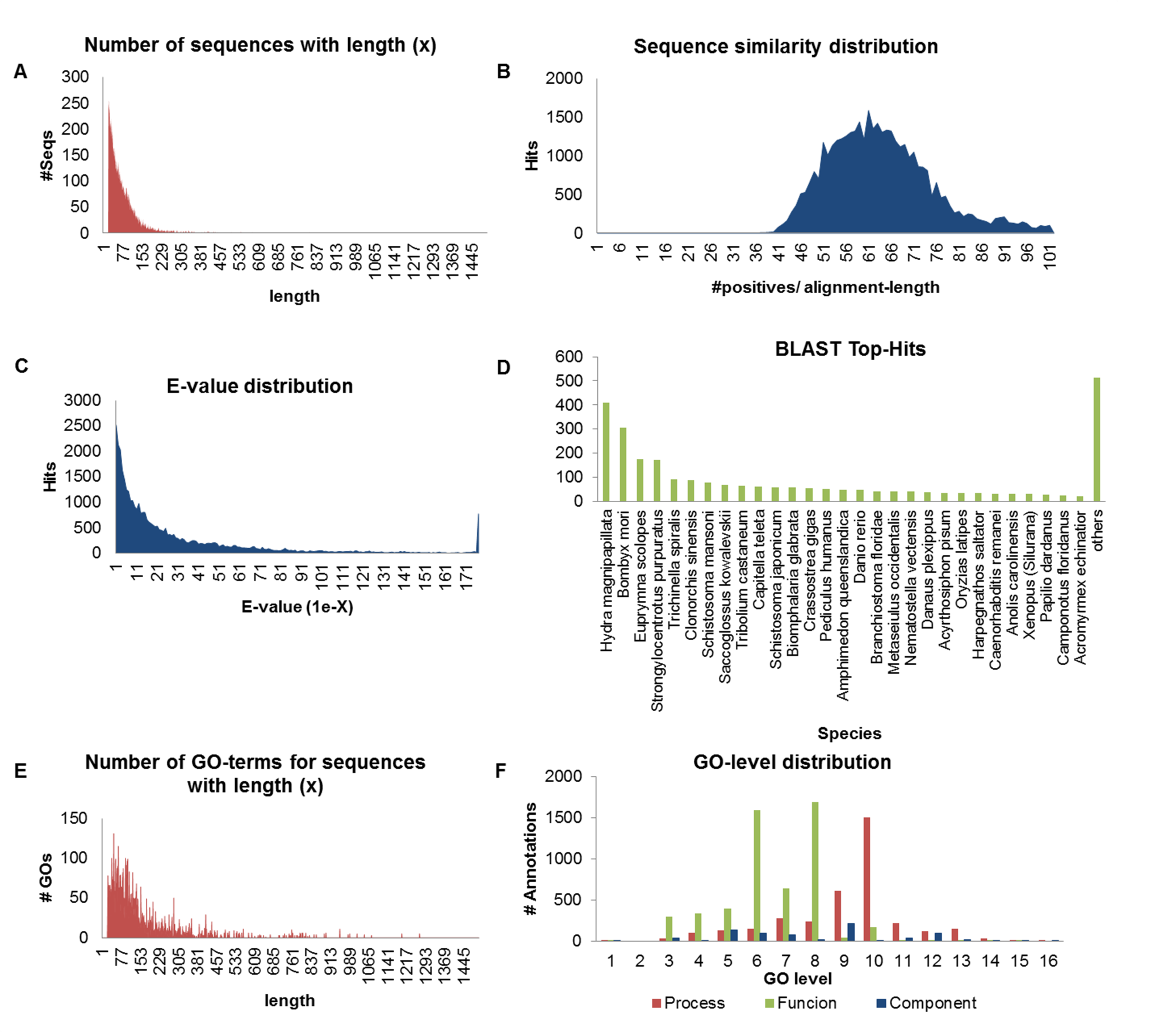

Supplement: S1 Fig — The transcriptome dataset was analyzed with Blast2GO software. (A) Length distribution of the assembled contigs. (B) Sequence similarity corresponding to BLAST hits. (C) Species distribution using BLASTx database. (D) Expected value distribution of sequence hits. (E) Number of GO terms assigned by sequences length. (F) Amount of GO levels assigned to annotated contigs based on gene ontology categorization. (TIF) [file pone.0119949.s001.tif]

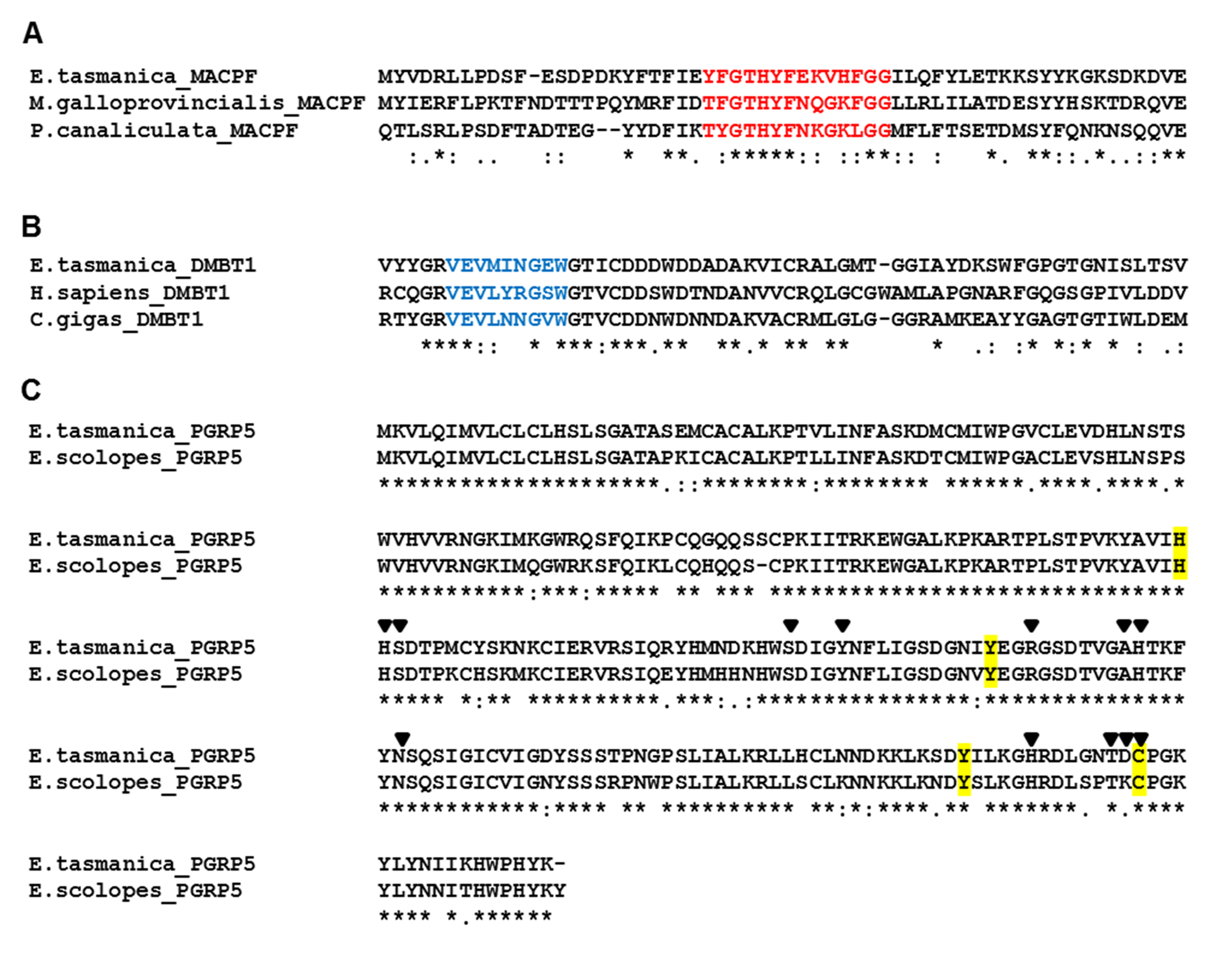

Supplement: S2 Fig — Newly identified immune transcripts in E. tasmanica were aligned with their close homologs protein sequences using ClustalX 2.1. A) Alignment of MAC perforin motifs (MACPF) is indicated in red. B) Comparison of DMBT1 protein sequences. VEVLXXXXW motif from the scavenger receptor cysteine rich domain (SRCR) in DMBT1 is marked in blue. C) E. tasmanica and E. scolopes peptidoglycan recognition protein 5 (PGRP5) comparison. The amino acids involved in the catalytic amidase site are highlighted in yellow. The arrowheads indicate substrate binding sites. For all alignments, asterisks indicate the conserved amino acids among proteins. GenBank accession numbers used in this figure: MACPF: Mediterranean mussel, Mytilus galloprovincialis (AEK10751); Aquatic snail, Pomacea canaliculata (P0C8G6); DMBT1: Pacific oyster, Crassostrea gigas (EKC27306); Human, Homo sapiens (NP_015568); PGRP5: Bobtail squid, Euprymna scolopes (AIR71819). (TIF) [file pone.0119949.s002.tif]
